# Environmental Pollutant PCB 153 Is Associated with Candidate Alternative Splicing Alterations in Intellectual Disability-Associated Genes: An Exploratory RNA-Seq Splicing Analysis in a Neuronal Model

**DOI:** 10.3390/genes17060692

**Published:** 2026-06-13

**Authors:** Maria Lui, Aurelio Minuti, Simone D’Angiolini, Michele Scuruchi, Serena Silvestro, Osvaldo Artimagnella

**Affiliations:** 1 IRCCS Centro Neurolesi “Bonino-Pulejo”, Via Provinciale Palermo, Contrada Casazza, 98124 Messina, Italy; maria.lui@irccsme.it (M.L.); serena.silvestro@irccsme.it (S.S.);; 2 Department of Clinical and Experimental Medicine, University of Messina, 98124 Messina, Italy

**Keywords:** polychlorinated biphenyls, alternative splicing regulation, RNA-binding proteins, computational splicing analysis, intellectual disability disorders

## Abstract

**Background/Objectives:** Polychlorinated biphenyls (PCBs) are persistent environmental contaminants associated with chronic toxicity and neurological dysfunction. PCB 153 is among the most prevalent congeners detected in environmental and dietary matrices. Although transcriptional responses to PCB 153 have been described, its potential association with post-transcriptional regulation remains poorly defined. Here, we performed an exploratory computational RNA-seq splicing analysis of previously generated transcriptomic data from retinoic acid-differentiated SH-SY5Y cells exposed to a sub-cytotoxic concentration of PCB 153. **Methods:** RNA-seq data were analyzed to identify candidate differentially alternative splicing events (DASEs). Candidate events were further examined for retained intron (RI)-related premature termination codons (PTCs), and potential regulatory interactions, including DASE-RNA-binding protein (RBP) motif enrichment. **Results:** PCB 153 exposure was associated with differential expression of 32 RNA-binding protein (RBP) encoding genes and with 90 candidate DASEs. Disease enrichment analysis indicates that genes affected by candidate splicing alterations overlapped with gene sets annotated to intellectual disability and related neurodevelopmental phenotypes. Among retained intron events, several were predicted to introduce PTCs, suggesting potential effects on transcript stability or coding potential. Motif enrichment analysis identified positional enrichment of motifs corresponding to CELF2, NUMA1, PRPF8, and RBM22 within DASE-associated regions, nominating these RBPs as putative regulators associated with the observed splicing alterations. **Conclusions:** This computational study identifies candidate PCB 153-associated splicing alterations and RBP-related regulatory hypotheses in a neuron-like in vitro model, suggesting a potential mechanistic link between PCB 153 and neurodevelopmental dysfunction.

## 1. Introduction

Polychlorinated biphenyls (PCBs) are a group of 209 man-made halogenated aromatic hydrocarbons characterized by a biphenyl backbone with between one and ten chlorine atoms attached [1]. PCBs were widely applied in numerous industrial uses, such as dielectric fluids in capacitors and transformers, as well as in pesticides, lubricants, and construction materials [2]. The long-term environmental stability and lipophilicity of PCBs promote their bioaccumulation in organisms and their accumulation in lipid-rich tissues and food matrices. Dietary PCB exposure is not restricted to fish-derived products: PCBs have also been detected in several high-fat foods of animal origin, including meat, dairy products, eggs, and fish, although the relative contribution of each food category varies according to dietary habits, geography, food-chain contamination, and monitoring practices [3]. Although PCB production was restricted and later banned in the U.S. in the 1970s due to health and environmental risks, PCBs are still unintentionally generated as byproducts during the productions of products like paints and silicone rubber [4,5]. It has been estimated that about 1.5 million metric tons of PCBs were produced worldwide, with nearly 10% of this amount persisting in the environment [6]. Among the various congeners, PCB 153 is one of the most detected in environmental samples [7]. According to a 2021 feed monitoring report from IMR (Bergen, Norway), PCB 153 was the most abundant PCB congener detected in fish feed (1.2 μg kg^−1^), fish meal (1.3 μg kg^−1^), and fish oil (4.8 μg kg^−1^) [8]. Regions with historical industrial PCB production, improper disposal, contaminated sediments, or high consumption of contaminated animal-derived foods may therefore represent contexts of increased exposure risk, although global comparisons remain limited by heterogeneous sampling and analytical approaches.

Numerous studies demonstrated that PCB exposure is correlated to higher rates of chronic diseases, such as cancer and endocrine, metabolic, and cardiovascular disorders [9,10,11,12]. Furthermore, epidemiological evidence indicated that exposure to environmental pollutants, such as PCBs, is associated with neurodegenerative diseases, including Parkinson’s disease (PD) [13], Alzheimer’s disease (AD) [14], and amyotrophic lateral sclerosis (ALS) [15]. PCBs have been detected in postmortem human brain samples [10], and higher concentrations of PCBs have been correlated with reduced cognitive function in aging populations [16].

Recent transcriptomic investigations have highlighted that exposure to PCB 153 elicits widespread molecular responses in differentiated SH-SY5Y cells, with pronounced effects on pathways involved in proteostasis and neurodegeneration [17]. These findings suggest that PCB 153 exposure is associated with early molecular alterations that may predispose neuronal cells to long-term dysfunction. While transcriptional and epigenetic regulation by PCBs has been widely documented [18,19], their association with AS remains poorly characterized. However, transcript abundance alone does not fully capture the complexity of gene regulation, where post-transcriptional mechanisms play a pivotal role in shaping cellular phenotypes.

Alternative mRNA splicing (AS) represents a fundamental post-transcriptional mechanism that enables the production of multiple mRNA variants from a single precursor transcript, thereby increasing protein diversity and playing a critical role in nervous system function [20]. Pre-AS is regulated by the spliceosome, a large macromolecular machinery composed of five small nuclear RNAs (U1, U2, U4, U5, and U6) and numerous associated proteins assembled into small nuclear ribonucleoproteins (snRNPs) [21]. RNA-binding proteins (RBPs) coordinate the formation of ribonucleoprotein complexes that control RNA processing, stability, localization, and translation [22]. Alterations in AS regulation, especially those mediated by RBPs, have been associated with several diseases, including neurodegenerative diseases [22,23]. Retained intron (RI) is a specific type of alternative splicing event (ASE) with significant regulatory consequences. Transcripts containing RIs frequently carry premature termination codons (PTCs), targeting them for degradation through nonsense-mediated decay (NMD) or potentially producing truncated or non-functional protein isoforms [24].

Building on our previously published transcriptomic dataset showing that PCB 153 exposure affects proteostasis and neurodegeneration-related pathways in RA-differentiated SH-SY5Y cells [17], the present study explores whether the same exposure condition is associated with candidate alterations in AS [25,26,27]. Using a computational RNA-seq splicing pipeline, we identified candidate differentially alternative splicing events (DASEs), examined retained intron events for predicted PTCs and potential NMD susceptibility, and evaluated whether genomic regions surrounding candidate DASEs were enriched for motifs of differentially expressed RBPs. Given the exploratory nature of the dataset, the findings are intended to prioritize molecular events and regulatory hypotheses for future experimental validation rather than to establish definitive causal mechanisms.

## 2. Materials and Methods

### 2.1. Reagents and Chemicals

PCB 153 was acquired from LGC Standards (Milan, Italy). Dimethyl sulfoxide, used for preparing stock solutions and treatments, was obtained from Sigma-Aldrich (Saint Louis, MO, USA). All chemicals were sterile filtered prior to use to prevent bacterial contamination.

### 2.2. Cell Culture and Experimental Treatments

The human SH-SY5Y neuroblastoma cell line was obtained from the American Type Culture Collection (ATCC; Manassas, VA, USA). Cells were seeded in 6-well plates (ThermoFisher Scientific, Rochester, NY, USA) at 5.0 × 10^5^ cells per well. Cultures were maintained in DMEM/F-12 Ham medium (Sigma-Aldrich, St. Louis, MO, USA) and incubated at 37 °C in a humidified atmosphere containing 5% CO_2_. Neuronal differentiation was initiated the day after seeding by treating cells with 10 μM RA (Sigma-Aldrich, St. Louis, MO, USA) for 5 days. Following differentiation, RA-treated SH-SY5Y cells were used as a simplified neuronal-like in vitro model, while acknowledging their neuroblastoma-derived origin, and exposed to varying concentrations of PCB 153 for 24 h.

Differentiated cells were exposed to 5 µM PCB 153 and the corresponding CTRL were analyzed in the present study. This concentration was selected based on our previous experiments [17], in which it was found to be non-cytotoxic but biologically active, inducing detectable transcriptomic responses without overt loss of cell viability. Therefore, 5 µM PCB 153 was used as a discovery-oriented condition to investigate early molecular perturbations under controlled in vitro conditions. We acknowledge that this concentration may exceed typical human exposure levels and should not be interpreted as directly equivalent to chronic environmental exposure scenarios.

### 2.3. RNA Isolation and cDNA Library Preparation

SH-SY5Y cells were plated in 6-well plates as previously described. After treatment, cells were detached using 0.25% trypsin-EDTA (Sigma-Aldrich, St. Louis, MO, USA) and collected by centrifugation at 300× *g* for 5 min. Total RNA was extracted from the resulting cell pellets using the Maxwell^®^ RSC simplyRNA Cells Kit (Promega, Madison, WI, USA) on the Maxwell^®^ RSC Instrument, following the manufacturer’s instructions. For library preparation, 100 ng of total RNA from two biological replicates was processed using the TruSeq^®^ RNA Exome Kit (Illumina, San Diego, CA, USA), according to the manufacturer’s protocol. Library quality was evaluated with the TapeStation 4150 system (Agilent, Santa Clara, CA, USA) using D1000 ScreenTape reagents (Agilent, Santa Clara, CA, USA). Prior to sequencing, libraries were denatured with 0.2 N NaOH and diluted to a final concentration of 1.42 pM. Sequencing was performed on an Illumina NextSeq™ 550Dx platform (Illumina, San Diego, CA, USA) using the NextSeq 500/550 Mid Output Reagent Kit v2.5 (150 cycles) in paired-end mode.

### 2.4. RNA-Seq and Transcriptome Analysis

Transcriptomic profiling of differentiated SH-SY5Y cells treated with PCB 153 compared with CTRL was previously conducted by our research group and reported by Minuti et al. [17]. To ensure the quality and reliability of the sequencing data, raw paired-end RNA-seq reads were first assessed using FastQC v.0.12.0 (Babraham Institute, Cambridge, UK) (FastQC; A Quality-Control Tool for High Throughput Sequence Data; available online: https://qubeshub.org/resources/fastqc, accessed on 3 February 2026). Low-quality bases and adapter sequences were subsequently removed with Trimmomatic v.0.40-rc1 (Usadel Lab, Aachen, Germany) [28]. The resulting high-quality reads were aligned to the human reference genome (GENCODE GRCh38/hg38 v39) using the STAR RNA-seq aligner v.2.7.10a STAR RNA-seq aligner v. 2.7.10a _alpha_220207 (New York, NY, USA) [29]. Gene-level transcript abundance was quantified from aligned reads with HTSeq v.0.13.5 [30]. Differential expression analysis was conducted in the R statistical environment (v.4.2.0, R Core Team) using DESeq2 (v.1.36.0) [31]. To control for multiple testing, *p*-values were adjusted using the Benjamini–Hochberg procedure, and genes with an adjusted q-value < 0.05 were considered differentially expressed. To assess the robustness of the dataset and the consistency of biological replicates, several quality-control analyses were performed. Variance-stabilized expression values were used for principal component analysis (PCA) and sample-to-sample distance assessment. Additional DESeq2 diagnostic analyses included dispersion estimation plots, MA plots, and Cook’s distance diagnostics. Because only two biological replicates per experimental condition were available, particular attention was given to the identification of potential outlier samples. No outliers were detected, and all samples were retained for downstream analyses.

To investigate post-transcriptional regulatory mechanisms potentially affected by PCB 153 exposure, we assessed differential expression of genes encoding RBPs, whose annotations were obtained from the catRAPID database [32]. Significantly differentially expressed RBPs were identified based on overlap between annotation and DESeq2 results and highlighted in a volcano plot to facilitate interpretation of PCB 153-associated changes in post-transcriptional regulation. The volcano plot was generated in Python using the Plotly library (v. 6.5.2) [33], plotting log_2_ fold change against −log_10_ adjusted *p*-values, with RBP genes emphasized in red and annotated directly on the figure.

### 2.5. Alternative Splicing Analysis

AS was characterized using rMATS, a computational tool designed to quantify and detect splicing differences between groups of RNA samples with replicates [34]. In this study, rMATS was applied to identify DASEs between PCB 153 and CTRL samples. Input files included BAM files, which are compressed binary forms of a SAM (Sequence Alignment Map), generated by STAR alignment against the GRCh38 reference genome [35], along with a GTF (Gene Transfer Format) file specifying gene structures and genome annotations. Analysis parameters were set to accommodate paired-end sequencing data (-t paired) and a mean read length of 75 base pairs (--readLength 75). Additionally, a minimum of 2 nucleotides was required at each end of splice junctions (--anchorLength 2), and detection of novel, unannotated splice sites was enabled (--novelSS). The rMATS output included SE, MXE, A5SS, A3SS, and RI events, with event-level estimates of Percent Spliced In (PSI), ΔPSI, and statistical significance. For each event, rMATS is calculated from the Percent Spliced In (PSI), reflecting the proportion of transcripts in which a particular exon is included. PSI values range from 0 (complete exon skipping) to 1 (exon always included) and are determined based on reads supporting either exon inclusion or exclusion. After PSI computation, the tool compared values between PCB 153 and CTRL samples to identify events showing statistically significant differences in exon inclusion.

To increase analytical stringency, only events with a false discovery rate (FDR) ≤ 0.05 were retained. Further filtering required a change in PSI (ΔPSI) of at least ±0.1. Additionally, low-confidence events with mean splice-junction coverage ≤ 5 were removed to reduce the influence of poorly covered junctions on PSI estimates.

### 2.6. Enrichment Analysis of DASE-Associated Genes

To investigate the functional and disease relevance of genes affected by DASEs in PCB 153 samples, enrichment analysis was performed using the enrich omics (v. 0.2.1) Python package (at https://github.com/saramasarone/enrich_omics, accessed on 2 February 2026). Enrich omics provides a Python interface to perform enrichment analysis and access multiple enrichment resources, including EnrichR [36], to identify overrepresented biological processes, pathways, and diseases for a given gene set.

EnrichR [36] is a widely used enrichment analysis tool that maintains numerous curated gene set libraries covering transcriptional regulation, canonical pathways, biological processes, cellular components, and disease associations. Using enrich_omics, we submitted the list of unique gene symbols corresponding to significant DASEs to EnrichR and retrieved enrichment results from the following libraries: DisGeNET (a curated resource integrating gene–disease associations derived from genetic studies, clinical reports, and literature mining [37]), KEGG 2021 Human (canonical pathways), GO Biological Process 2025, GO Molecular Function 2025, and GO Cellular Component 2025. EnrichR calculates statistical significance using adjusted *p*-values, Z-scores, and combined scores, providing quantitative measures of enrichment. Terms with an adjusted *p*-value ≤ 0.05 were considered significant.

### 2.7. RI Premature Stop Codon Identification

RI events may introduce PTCs occurring upstream with respect to the canonical transcript termination site [38]. Since our analysis focused on genes associated with ID, RI events were first filtered to retain only those involving genes enriched for this disease in the DisGeNET database [39].

To identify RI events potentially generating PTCs in the PCB 153 vs. CTRL comparison, genomic coordinates corresponding to the retained intron were extracted and extended by two nucleotides upstream and downstream to preserve codons spanning exon–intron junctions. The resulting intervals were formatted as BED files and used as input for sequence extraction with the getfasta utility from the BEDTools suite (v2.30.0) [40]. Sequences were retrieved from the human reference genome GRCh38 downloaded from Ensembl [35] and exported in FASTA format while preserving strand orientation. To determine the translational context of each RI event, genomic loci were manually inspected using the Integrative Genomics Viewer (IGV) [41], allowing identification of coding sequence boundaries and manual assignment of the reading frame (frame 1, 2, or 3) at the RI junction.

To evaluate whether RI inclusion could introduce premature stop codons, we implemented an in-house Python script (Python v3.9.12) using the Biopython package (v.1.84) [42]. RI nucleotide sequences were translated in the manually assigned reading frame without truncation at the first stop codon, enabling detection of all in-frame termination codons. Stop codons (TAG, TAA, or TGA) were recorded together with their nucleotide positions within the RI sequence. This workflow enabled systematic identification of RI events predicted to introduce premature termination codons, thereby nominating transcripts potentially susceptible to NMD or altered coding potential.

### 2.8. RBPbench Motif Analysis of Differentially Expressed RBP on DASE Sequences

RBP motif analyses were performed using RBPBench, an open-source computational tool for detection, enrichment, and co-occurrence analysis of RBP motifs (available at: https://github.com/michauhl/RBPBench, accessed on 2 February 2026). Genomic regions associated with DASEs were separated into two BED files representing the positive-ΔPSI and negative-ΔPSI subsets, respectively. Each subset was analyzed independently. For both sets, we first applied the ENMO mode of RBPBench to evaluate motif enrichment and co-occurrence statistics and then applied the SEARCH mode to obtain position-specific motif occurrences within DASE regions. In both analyses, the motif enrichment and search were restricted to the subset of 32 RBPs that were also differentially expressed in our comparison.

RBPBench ENMO analysis evaluated whether motif occurrences for selected RBPs were significantly over- or under-represented in the input regions relative to a sampled genomic background and computed single-motif enrichment statistics using Fisher’s exact test with multiple-testing correction. Specifically, BED regions were analyzed against the GRCh38 reference genome FASTA and gene annotations from Ensembl (GRCh38.112 GTF). Motif scanning was performed internally by RBPBench using FIMO with a stringent significance threshold. Input regions were extended by ±250 nucleotides to include flanking sequences, and enrichment calls were filtered using an adjusted *p*-value threshold of 0.05. Both enrichment/depletion and motif co-occurrence analysis were enabled together with appropriate multiple-testing corrections (with Benjamini–Hochberg).

Following enrichment analysis, we then used RBPBench with SEARCH mode on the same region sets, to identify RNA-binding protein motif occurrences within the corresponding genomic intervals. The SEARCH mode was applied to obtain coordinate-resolved maps of motif occurrences within DASE regions. The same genome, transcript annotations, RBP list, motif-scanning threshold, and flanking window size were used to ensure consistency with the ENMO analysis. SEARCH outputs included UpSet plots illustrating motif set intersections and interactive HTML motif visualizations. Wilcoxon rank-sum tests were used to compare motif scores between regions with and without motif hits.

This workflow enabled the identification of enriched motifs for each differentially expressed RBP within DASE genomic regions and the determination of their precise genomic positions. Motif enrichment results from ENMO analysis were integrated with coordinate-resolved motif occurrences obtained from the SEARCH module, allowing localization of significantly enriched motifs for each RBP. Enriched motifs were therefore annotated for their genomic position and classified as upstream, downstream, or exonic, with upstream and downstream regions defined as the 250-nucleotide flanking windows used during motif scanning. Motif distributions across DASE regions (upstream, exon, or downstream) were subsequently visualized using a custom pipeline implemented in Python (v3.9.12), using Pandas (v2.2.2) for data processing, Seaborn (v0.13.2) and Matplotlib (v3.9.1). This approach was used as a candidate prioritizing strategy and does not demonstrate direct RBP binding or functional regulation.

## 3. Results

### 3.1. PCB 153 Exposure Is Associated with Differential Expression of RBP-Encoding Genes in RA-Differentiated SH-SY5Y Cells

In our previous work, we investigated the transcriptomic response of RA-differentiated SH-SY5Y neuronal cells exposed to a sub-cytotoxic concentration of PCB 153 equal to 5 μM [17]. Here, we leveraged this RNA-seq data to explore whether the contaminant also affects post-transcriptional regulation. To this end, we first examined whether genes encoding RBPs, which play central roles in RNA processing and splicing regulation [22], were differentially expressed between PCB 153-treated cells and CTRL. Quality-control analyses confirmed the robustness of the RNA-seq dataset despite the limited number of biological replicates. PCA of variance-stabilized expression data showed clear clustering of biological replicates and separation between experimental groups (Figure S1). In addition, DESeq2 dispersion estimates, MA plots, and Cook’s distance diagnostics supported the reliability of the statistical model and did not identify influential observations or outlier samples (Figures S2–S4).

**Figure 1:**
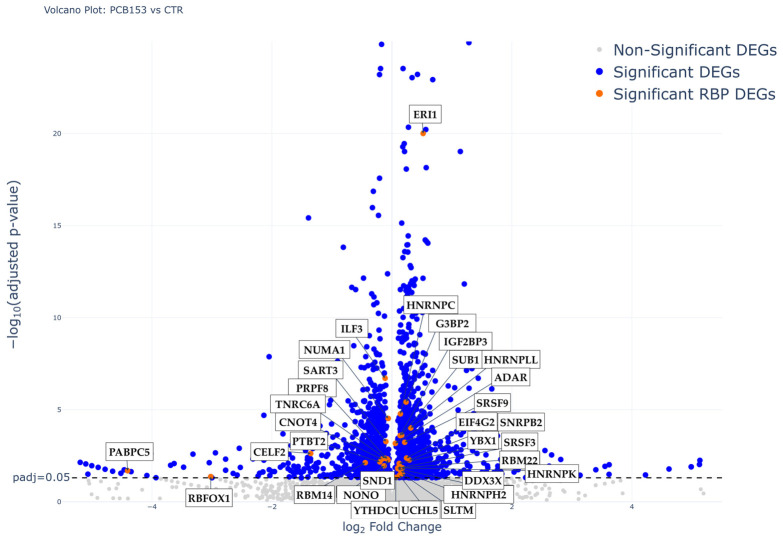
Volcano plot highlighting differentially expressed RBPs in PCB 153 vs. CTRL. All genes from the differential expression analysis are displayed according to log2 fold change on the x-axis and −log10(q-value/FDR-adjusted *p*-value) on the y-axis. Genes with adjusted *p*-value < 0.05 are shown as significant in blue, while non-significant genes are shown in gray. RBPs are highlighted in orange. RBPs were identified using the catRAPID database, a curated reference set of experimentally characterized RNA-binding proteins used within the catRAPID framework [32]. The horizontal dashed line represents the adjusted *p*-value threshold of (*p* < 0.05). The figure was generated in Python using the Plotly library.

Among the 1882 significantly differentially expressed genes (DEGs), 32 were found to encode for RBPs (Table S1), according to annotations from the catRAPID (v. 2.1) database [32], indicating that PCB 153 exposure may affect post-transcriptional regulatory pathways (Figure 1). Notably, three RBPs (PABPC5, RBFOX1, and CELF2) were strongly downregulated (log_2_ Fold Change < −1). RBFOX1 and CELF2 are well-established splicing regulators [43,44], while PABPC5 is known to bind the poly(A) tail of eukaryotic mRNAs and regulate nuclear–cytoplasmic export, transcript stability, and translation [45], processes frequently linked to splicing-associated regulatory mechanisms [46,47]. Together, these observations indicate that PCB 153 exposure was associated with differential expression of a subset of RBPs, including known splicing regulators. This suggests that PCB 153 exposure may influence gene expression by modulating post-transcriptional mechanisms, particularly those governing AS.

### 3.2. PCB 153 Exposure Is Associated with Candidate Differential Alternative Splicing Events

**Figure 2:**
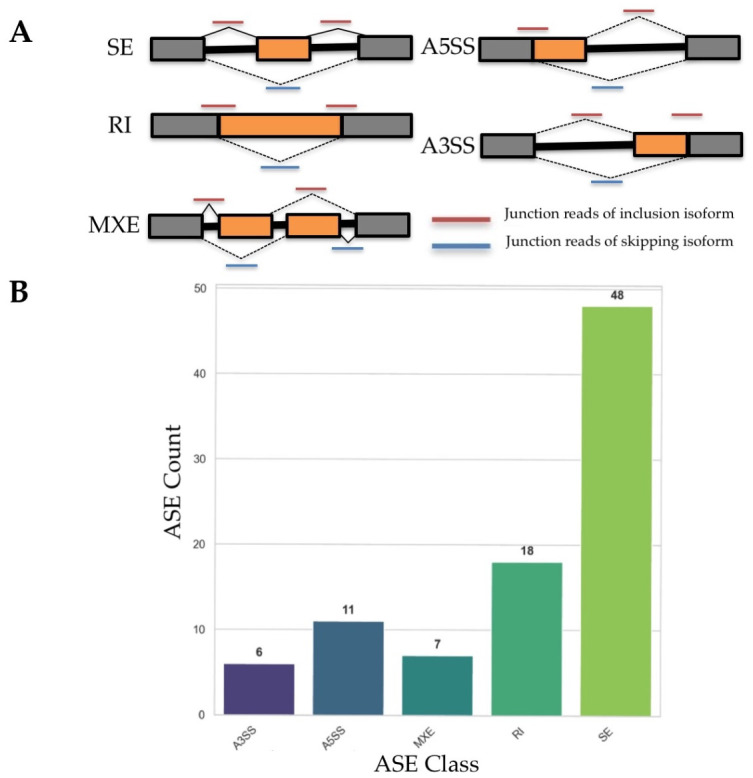
rMATS analysis of DASE identification for PCB 153 vs. CTRL comparison. (**A**) Schematic illustration of rMATS method for detecting ASEs [48,49], designed to make it easier to interpret the effects of ASEs on exon and intron usage, which can otherwise be complex to understand. Five major types of events are shown: A3SS, A5SS, MXE, RI, and SE. In each schematic representation of a splicing event, alternatively spliced exons or introns are highlighted in orange, while constitutive flanking exons are shown in gray. Red arcs represent splice-junction reads supporting inclusion of an isoform, whereas blue arcs indicate junction reads supporting a skipping isoform. These junction read patterns are used by rMATS to classify splicing events. (**B**) Counts of ASEs per class for differentiated SH-SY5Y treated with PCB 153 versus untreated differentiated SH-SY5Y. Bars indicate the number of ASEs in each class, with numeric values shown above each bar. Only statistically significant DASEs identified by rMATS (FDR ≤ 0.05, absolute value of ΔPSI ≥ 0.1, mean junction coverage > 5) were included in the analysis. Plot generated in Python (v3.9.12) using pandas (v2.2.2), Seaborn (v0.13.2), and Matplotlib (v3.9) from the rMATS output.

To determine whether the observed changes in RBP expression were associated with global alterations in splicing patterns, we examined the impact of PCB 153 exposure on AS by comparing RA-differentiated SH-SY5Y cells treated with 5 µM PCB 153 to untreated CTRL. To comprehensively detect and quantify differential DASEs, we applied replicate Multivariate Analysis of Transcript Splicing (rMATS) turbo v4.3.0 as part of a standardized analytical pipeline [34]. Under the applied rMATS filtering criteria, we identified 90 candidate DASEs in PCB 153-treated versus control cells, including six alternative 3′ splice sites (A3SS), 11 alternative 5′ splice sites (A5SS), seven mutually exclusive exons (MXE), 18 retained introns (RI), and 48 skipped exon (SE) events (Figure 2, Table S2).

### 3.3. Candidate PCB 153-Associated DASE Genes Are Enriched for Intellectual Disability-Related Disease Annotations

To investigate the potential pathological and functional relevance of candidate PCB 153-induced splicing alterations, we performed enrichment analyses using Gene Ontology annotations, KEGG pathways, and disease associations from DisGeNET. Functional enrichment analysis of genes associated with significant DASEs revealed no significant enrichment for canonical pathways, biological processes or cellular components; instead, the analysis showed a significant enrichment for molecular function terms and disease annotations. Complete results are reported in Table S3 (molecular function and disease enrichment) while summarized enrichment results are given in Table 1.

Disease enrichment analysis using DisGeNET identified “Intellectual Disability” (ID) as the most significantly enriched disease term based on adjusted *p*-value. The genes contributing to this enrichment included *ABCD4*, *KMT2A*, *DCTN1*, *HTRA2*, *VLDLR*, *AFF4*, *OPA1*, *FLAD1*, *NSD1*, *KMT5B*, *ACADM*, *TXNL4A*, *CTSA*, *RUBCN*, *ADSL*, *STRADA*, *UROS*, *TINF2*, *LAMB1*, *GDAP1*, *POMGNT1*, *CLK2*, *DCX*, *RAB3GAP2*, *TAF1*, and *FGFR1*. These enrichments suggest that the genes associated with candidate DASEs overlap with gene sets previously annotated to neurodevelopmental disorders, particularly ID. To note, two of them also enriched the “Histone H4K20 Methyltransferase Activity” term.

**Figure 3:**
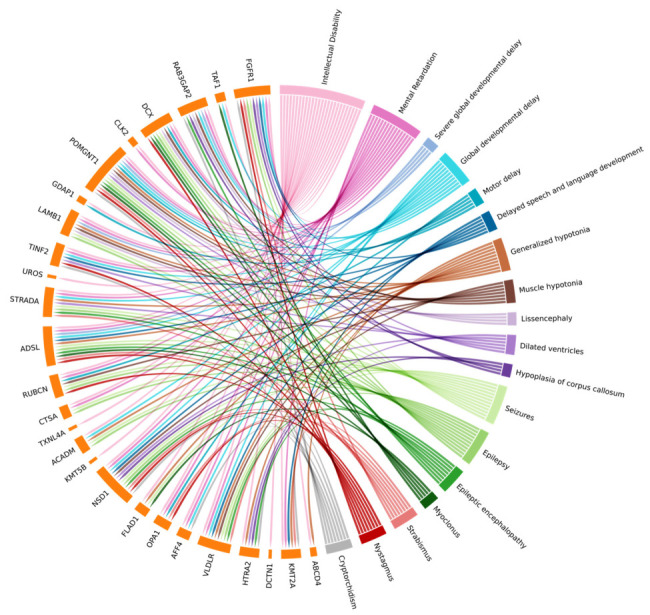
Chord plot of DASE-enriched genes associated with ID-related phenotypes, generated using the Python d3blocks package. Arcs represent either genes or phenotypes, and chords connect each gene to the phenotypes it enriches. The plot was produced by first filtering for overlapping genes between ID and related phenotypes, then mapping each gene–phenotype association as a chord. Color coding differentiates phenotypes and highlights patterns of shared genetic contributors across multiple disorders.

To evaluate whether the other significantly enriched diseases were linked to ID, we queried DisGeNET for disorders annotated as associated with ID. We then filtered our list of enriched disease terms for those associated with ID. This analysis identified 17 enriched diseases that are also linked to ID. These included the DisGeNET term “Mental Retardation”, developmental delay related conditions (“Global developmental delay”, “Severe global developmental delay”, “Motor delay”, and “Delayed speech and language development”), neurological manifestations (“Seizures”, “Epilepsy”, “Epileptic encephalopathy”, “Myoclonus”, “Lissencephaly”, “Hypoplasia of the corpus callosum”, and “Dilated ventricles”), neuromuscular features (“Generalized hypotonia” and “Muscle hypotonia”), and additional clinical traits such as “Strabismus”, “Nystagmus”, and “Cryptorchidism”. Each of these terms represents a unique entry within the DisGeNET database; however, “Mental Retardation” is not conventionally used in clinical practice, as it is usually referred to as “Intellectual Disability” and does not typically represent a distinct disease with separate, distinctive symptoms. Nonetheless, for completeness and reproducibility purposes, we maintained the original annotations included in the DisGeNET database. Together, these findings indicate that genes affected by candidate differential AS overlap with a broad spectrum of neurodevelopmental and neurological disease annotations. Focusing on genes associated with ID, we next assessed whether these genes also contributed to the enrichment of related phenotypes. Gene–disease relationships were visualized using a chord diagram (Figure 3), which highlights extensive sharing of genetic contributors across multiple ID-related phenotypes. This shared genetic architecture suggests that candidate PCB 153-associated splicing alterations occur in genes previously linked to convergent neurodevelopmental phenotypes (Figure S5). However, because DisGeNET-based enrichment reflects curated gene–disease associations and not direct functional validation in this model, these findings should be interpreted as prioritization signals for future mechanistic studies rather than evidence of disease-causative disruption.

### 3.4. PCB 153 Exposure Was Associated with Production of Non-Functional Proteins

**Figure 4:**
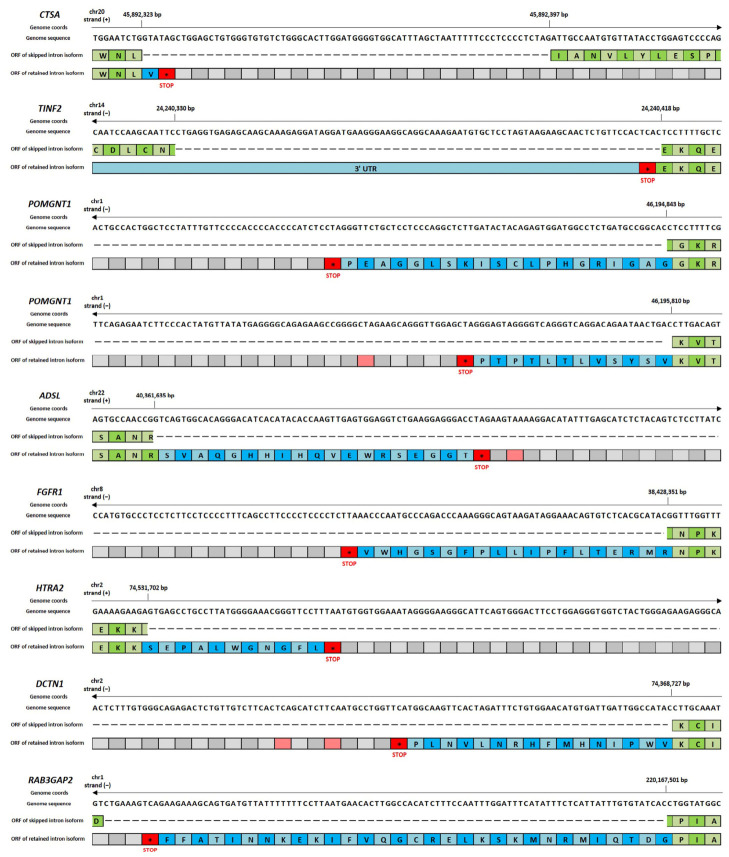
All events listed in the table were manually inspected using IGV and are shown as representative examples of PTC identification in RI DASEs. Asterisk symbols in red boxes were used to indicate translation stop codons. Green boxes represent amino acids coded by canonical isoform, whereas the blue ones represent amino acids or the 3′UTR of the alternative isoform. Grey boxes indicate untranslated codons in the alternative isoforms.

To assess the potential functional impact of RI events in genes associated with ID, we analyzed them for the presence of PTCs in PCB 153-treated vs. CTRL samples. Most RIs were predicted to generate one PTC (Table 2 and Table S4), with some events introducing multiple PTCs, such as *ADSL* (36 predicted PTCs), *POMGNT1* (17 predicted PTCs), and *DCTN1* (10 predicted PTCs). The position of the first PTC within each RI sequence was also annotated (Table 2), allowing a preliminary evaluation of compatibility with the canonical NMD rule (>50–55 nucleotides upstream of the last exon–exon junction) [50]. Only *RAB3GAP2* did not satisfy the NMD rule, suggesting that this RI will generate a truncated protein. Interestingly, in the case of *TINF2*, the PTC identified seems in-frame with the 3′ untranslated region (UTR) of a known shorter *TINF2* coding isoform. The ΔPSI values were predominantly negative, with the largest reductions observed for *CTSA* (ΔPSI = −0.357), *TINF2* (ΔPSI = −0.208), and *POMGNT1* (ΔPSI = −0.190/−0.241), while *RAB3GAP2* displayed a positive ΔPSI (0.197). Graphical representation of PTCs identification is shown in Figure 4, with canonical amino acids in green, alternative isoform-encoded residues in blue, and PTCs highlighted with red asterisks.

### 3.5. Candidate RBP Motifs Show Positional Enrichment Across DASE-Associated Genomic Regions

We investigated the positional distribution of RBP motifs across ID-related DASEs using RBPBench tool (v1.0.1). We analyzed motif occurrences for 32 RBP DEGs within DASE genomic regions. Among these, four RBPs (CELF2, NUMA1, PRPF8, and RBM22) exhibited motifs that were significantly overrepresented. The full list of motif occurrences, including genomic coordinates, splicing event class, associated genes, significance (FDR), and motif positions, is provided in Table S5.

**Figure 5:**
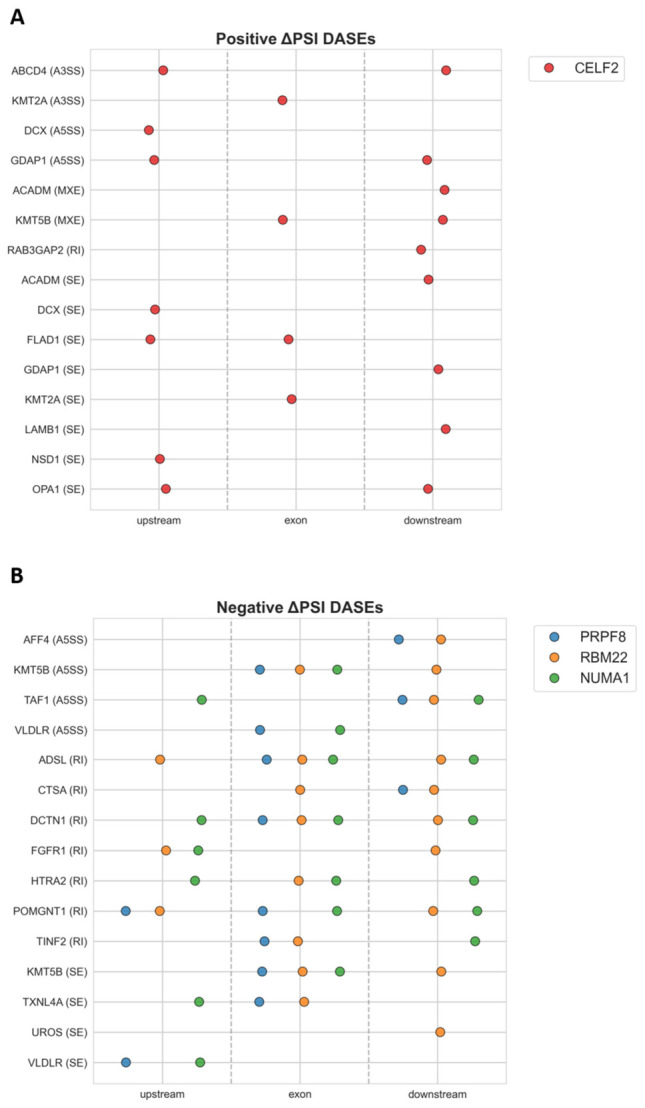
(**A**) Distribution of RBP binding motifs across AS regions for DASEs with positive inclusion level changes (ΔPSI > 0). (**B**) Distribution of RBP binding motifs across AS regions for DASEs with negative inclusion level changes (ΔPSI < 0). In both panels, each point represents a unique gene–event combination, labeled by gene symbol and splicing event class. Motifs are grouped by their position relative to the regulated exon and colored according to the corresponding RNA-binding protein. Vertical dashed lines indicate boundaries between positional categories. Only motifs identified as significantly enriched by RBPBench enrichment analysis (Fisher’s exact test with Benjamini–Hochberg correction, adjusted *p*-value ≤ 0.05) were included in the visualization. The plot was produced using Seaborn (v0.13.2), Pandas (v2.2.2), and Matplotlib (v3.9.1) in Python, with jittering applied to separate overlapping points, illustrating positional trends of RBP binding associated with decreased exon inclusion.

**Figure 6:**
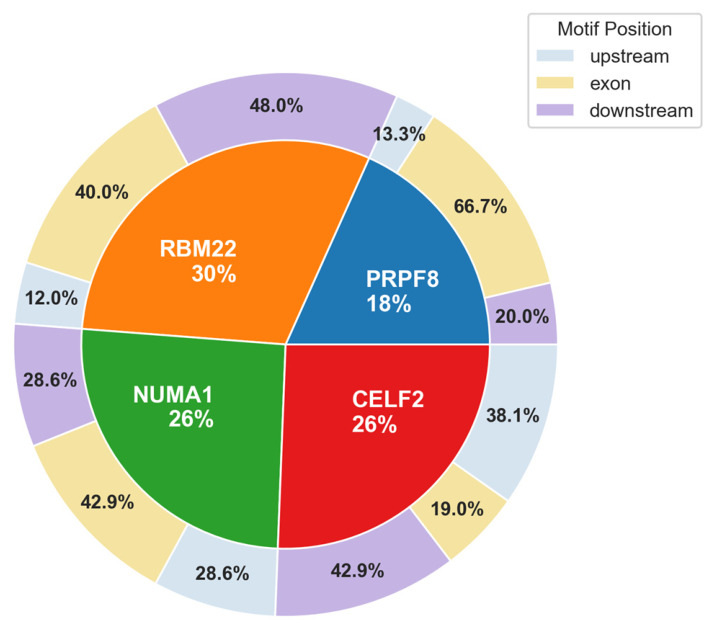
Nested pie chart showing the distribution of RBP binding motifs across AS regions. Only motifs from RBPs significantly enriched in the RBPBench analysis (adjusted *p*-value ≤ 0.05) are shown. The inner ring represents the total number of motif occurrences per RBP (PRPF8, RBM22, NUMA1, CELF2) with percentage labels, while the outer ring shows the relative distribution of motifs by position (upstream, exon, downstream) for each RBP. Each wedge in the outer ring is labeled with the percentage of motifs relative to its RBP total. The figure was generated in Python using NumPy (v1.26.6), Pandas (v2.2.2), and Matplotlib (v3.9.1). Legend indicates motif positions. The nested pie visualization highlights positional enrichment patterns of RBP motifs across regulated exons.

Analysis revealed distinct positional patterns associated with ΔPSI values (Figure 5 and Figure 6). For DASEs with positive ΔPSI, CELF2 motifs were the only significantly enriched motifs, predominantly located upstream and downstream of regulated exons. For DASEs with negative ΔPSI, motifs for NUMA1, PRPF8, and RBM22 were significantly enriched. PRPF8 motifs were most frequently located within exons; RBM22 motifs were mostly enriched in exons and downstream regions, and NUMA1 motifs were primarily found in all three regions.

Overall, these results provide a high-resolution map of enriched RBP motifs across candidate DASE-associated regions. Although motif localization may suggest sequence contexts compatible with RBP-mediated regulation, these data do not demonstrate direct RBP binding or functional regulation. Therefore, CELF2, NUMA1, PRPF8, and RBM22 should be interpreted as candidate RBPs for future validations.

## 4. Discussion

In this study, we performed an exploratory computational analysis of previously generated RNA-seq data to investigate whether PCB 153 exposure is associated with candidate alterations in AS in RA-differentiated SH-SY5Y cells. The analysis identified candidate DASEs, and predicted RI/PTC consequences and motif enrichment patterns involving differentially expressed RBPs. These findings suggest that post-transcriptional regulation may represent an additional molecular layer associated with PCB 153 exposure. Interestingly, a disease enrichment analysis using the DisGeNET database [39] revealed that PCB 153-regulated DASEs are significantly associated with genes linked to specific pathological conditions correlated with ID. It is a common neurodevelopmental disorder characterized by limitations in both intellectual functioning, such as reasoning and problem solving, and adaptive functioning (conceptual, social, and practical skills). These deficits emerge during the developmental period [51], and frequently co-occur with other neurodevelopmental and neuropsychiatric conditions, including autism, schizophrenia, and epilepsy [52].

The chord plot analysis further highlighted that several DASE-enriched genes are shared across multiple neurodevelopmental phenotypes, suggesting convergence toward common molecular pathways critical for brain development and function. Importantly, while DisGeNET identifies gene–disease associations, it does not systematically distinguish whether the underlying pathogenic mechanism is due to loss of function, gain of function, dominant negative effects, haploinsufficiency, or altered dosage sensitivity [53].

The relevance of splicing regulation to neurodevelopmental disorders is further supported by recent discoveries involving non-coding spliceosomal RNA genes. De novo variants in RNU4-2, which encodes U4 snRNA, have been identified as a frequent cause of syndromic neurodevelopmental disorders characterized by intellectual disability and developmental delay [54,55]. Similarly, recurrent variants in RNU2-2, encoding U2 snRNA, have been associated with a severe neurodevelopmental disorder with prominent epilepsy [56]. In addition, alterations affecting RNU12, a component of the minor spliceosome, have been linked to defective minor intron splicing and neurological disease phenotypes, including early-onset cerebellar ataxia and CDAGS syndrome [57,58]. Although the present study does not investigate pathogenic variants in snRNA genes, these findings reinforce the concept that perturbation of spliceosomal components and splicing regulation can have major consequences for neurodevelopmental biology.

To better define the functional impact of these alterations, we evaluated the PTC-generating RI events in ID associated genes, and the assessment of RBP motif distribution within ID-related DASEs. RI events that introduce a PTC are predicted to generate transcripts recognized by the NMD machinery. During splicing, if an RI introduces a PTC more than 50–55 nucleotides upstream of the final exon–exon junction, the presence of a multiprotein complex known as the exon junction complex (EJC) promotes NMD-mediated degradation [46]. This mechanism couples splicing fidelity to mRNA surveillance and represents a crucial quality-control system controlling transcript stability [59]. Before producing significant amounts of truncated protein, RI acts as a post-transcriptional regulator by destabilizing transcripts [60]. The direction of ΔPSI change provides functional insight into transcript fate. Negative ΔPSI values for PTC-generating RIs indicate reduced intron inclusion, suggesting a shift away from NMD-targeted isoforms and toward more stable, potentially translatable transcripts. In contrast, positive ΔPSI values reflect increased intron retention, promoting NMD-mediated decay.

In our model, almost all predicted PTC-generating RI events displayed negative ΔPSI values, with *RAB3GAP2* representing the only highlighted exception. Reduced intron retention was observed in a subset of prioritized RI/PTC candidate events involving *CTSA*, *TINF2*, *POMGNT1*, *ADSL*, *FGFR1*, *HTRA2*, and *DCTN1* (RI event IDs: 574, 2180, 4044/4046, 4378, 4980, 6039, and 6381, respectively; ΔPSI range: −0.357 to −0.121; FDR range: 0.0133–0.0497; Supplementary Table S2). Predicted premature stop codons for these events are reported in Supplementary Table S4. Conversely, *RAB3GAP2* showed increased intron retention (RI event ID: 7029; ΔPSI = +0.197; FDR = 0.0240; Supplementary Table S2), with a predicted PTC reported in Supplementary Table S4. However, this event did not meet the canonical NMD rule, suggesting that the corresponding candidate transcript could theoretically escape NMD; importantly, this remains a sequence-based prediction and does not demonstrate NMD escape, transcript stability changes, or truncated protein production.

The *CTSA* gene belongs to the serine protease family, a multifunctional glycoprotein with three distinctive hydrolytic activities [61]. Beyond its canonical lysosomal role, cathepsin family members have been implicated in synaptic plasticity, neural progenitor regulation, and extracellular matrix remodeling within the nervous system [62,63]. However, cathepsin activity must be carefully regulated, as both deficiency and overactivity can result in pathological consequences. Deficient cathepsin function has been linked to the buildup of toxic protein aggregates, impaired lysosomal activity, and neuronal cell death [64]. Conversely, lysosomal membrane permeabilization-induced leakage of cathepsins into the cytosol may activate apoptosis or inflammatory signaling, resulting in neurotoxic effects [65].

Interestingly, *TINF2* encodes one of the proteins of the shelterin, or telosome, complex which protects telomeres by allowing the cell to distinguish between telomeres and regions of DNA damage. In the present study, *TINF2* displayed a predicted PTC-generating RI event with negative ΔPSI, promoting the coding of the long *TINF2* isoform. It has been shown that overexpression of *TINF2* led to telomere shortening [66]. Mutations in *TINF2* are associated with dyskeratosis congenita and developmental abnormalities [67]. Functional studies have demonstrated that suppression of *TINF2*, either through pathogenic mutant alleles or RNA interference, results in telomerase-mediated telomere over-elongation. In contrast, ectopic overexpression of *TINF2* in human cell lines restricts telomere extension [68,69].

*POMGNT1* encodes a type II transmembrane protein localized to the Golgi apparatus, where it participates in O-mannosyl glycosylation, specifically targeting alpha-linked terminal mannose residues [70]. In AD models, reduced *POMGNT1* expression has been reported [71], suggesting that impaired glycosylation pathways may contribute to neurodegenerative processes. However, accumulating evidence indicates that *POMGNT1* activity is highly context dependent. In glioblastoma multiforme, the most invasive and aggressive brain tumor, elevated *POMGNT1* expression has been associated with a poor prognosis [72].

*FGFR1* encodes a transmembrane catalytic receptor with intracellular tyrosine kinase activity, belonging to a family of receptors bound by fibroblast growth factors (FGFs). FGFs are extracellular signaling molecules that regulate cell proliferation, differentiation, and migration during embryonic development [73]. FGFR1 is expressed in several tissues, including the brain, acting mainly via PI3K and MAPK pathways [74,75,76]. Genetic variants of the *FGFR1* gene determine the so-called “*FGFR1*-related Hartsfield syndrome”, characterized by malformations (such as craniofacial dysmorphism) and neurologic issues (such as developmental delay and seizures) [77,78]. *FGFR1* upregulation has been reported to be a critical oncogenic driver in different cancers [75,79]. Although FGFR1 dysregulation has been implicated in oncogenic contexts, the present data does not allow any inference regarding carcinogenic effects of PCB 153 in this model.

The *HTRA2* gene encodes a serine protease localized in the endoplasmic reticulum and mitochondria with release to the cytosol following apoptotic stimulus. Indeed, HTRA2 induces apoptotic cell death by two distinct mechanisms: the removal of BIRC-dependent suppression of caspases, resulting in increased caspase activity, and a BIRC- and caspase-independent pathway that depends on its serine protease function [80]. Variants in the *HTRA2* gene were associated with an early-onset mitochondrial syndrome characterized by seizures, hypotonia, and cardio-respiratory problems [81]. Upregulation of HTRA2 protein has been reported to induce mitochondrial defects and neurodegeneration in mice via induction of apoptosis in the brain [82].

*DCTN1* encodes the dynactin subunit 1, part of the dynactin complex. It has a central function in dynein-dependent retrograde trafficking of vesicles and organelles along microtubules by facilitating the recruitment of dynein to the microtubule network [83]. Moreover, dynactin is also involved in chromosome movement [84], and axon genesis [85]. Mutations in this gene originate from the spectrum of *DCTN1*-related neurodegeneration, which includes Perry syndrome, distal hereditary motor neuronopathy type 7B, frontotemporal dementia, motor neuron disease/ALS, and progressive supranuclear palsy. Some individuals present overlapping phenotypes [86].

The *RAB3GAP2* gene has the highest level of expression in the brain and encodes a protein belonging to the RAB3 protein family. It is involved in neurotransmission and neuronal activity, regulating vesicle trafficking and exocytosis [87]. Specifically, RAB3GAP2 is the regulatory subunit of the Rab3 GTPase-activating complex interacting with RAB3GAP1, which constitutes the catalytic subunit [88]. It is also important during neurodevelopment [88]. Indeed, mutations in this gene are associated with Martsolf syndrome, characterized by congenital cataracts, hypogonadism, and mild ID [89,90]. Moreover, emerging evidence indicates that dysregulation of Rab3 proteins is linked to age-associated neurodegenerative disorders, including AD, PD, and Huntington’s diseases. In this regard, altered Rab3 function has been associated with defective autophagy, synaptic impairment, and neuronal loss [88]. Therefore, *RAB3GAP2* represents a biologically relevant candidate for future validation of RI/PTC consequences in neurodevelopmental and neurodegenerative contexts.

Regarding the enrichment of motifs corresponding to the 32 differentially expressed RBPs, *CELF2* motifs were enriched among DASE-associated regions with ΔPSI > 0, whereas *RBM22*, *NUMA1*, and *PRPF8* motifs were enriched among DASE-associated regions with ΔPSI < 0 (Supplementary Table S5). These motif enrichment results were interpreted together with RBP expression changes reported in Supplementary Table S1, where *CELF2*, *NUMA1*, and *PRPF8* showed reduced expression, while *RBM22* was upregulated after PCB 153 exposure. To improve traceability, the complete list of DASE-associated regions carrying enriched RBP motifs, together with event class, gene symbol, FDR, ΔPSI, motif ID, and motif position, is provided in Supplementary Table S5. Specifically, CELF2 is an RBP belonging to the CELF/BRUNOL protein family, which is involved in regulation of pre-mRNA alternative splicing. Interestingly, CELF2 mutations and deficiency have been linked to neurodevelopmental disorders, including autism spectrum disorder (ASD) [91,92], and with epileptic manifestations [66], corroborating our results on its RNA-binding prediction on genes related to ID.

PRPF8 is a core component of the spliceosome machinery, having scaffolding roles in pre-catalytic, catalytic and post-catalytic functions of the pre-mRNA splicing process [93]. Mutations in this gene are a recognized cause of autosomal dominant retinitis pigmentosa [94]. Recently, variants in the *PRPF8* gene were detected in patients characterized by ID and autistic behaviors [95,96]. In this view, our results on PRPF8 motif enrichment of ID DASEs seem to corroborate its involvement in neurodevelopmental disorders. Moreover, downregulation of the *PRPF8* gene is linked to a variety of cancers, inducing abnormal splicing events [97]. Interestingly, PRPF8 was found in association with RBM22 RBP within human spliceosome machinery [98].

The *NUMA1* gene encodes for the nuclear mitotic apparatus protein 1, which is first characterized as a player during cell division, interacting with microtubules to organize the mitotic spindle for the segregation of chromosomes [99,100]. During differentiation, NUMA1 has been described to be involved in chromatin organization, splicing factor speckle distribution, as well as DNA damage response and apoptosis upon cellular stress [101]. Recently, it has been reported that in post-mitotic neurons NUMA1 is transiently translocated to the axon initial segment (the site where action potential starts), promoting its assembly and stability [102]. Interestingly, downregulation of it was found in Huntington’s disease models generating defects in microtubule networks and axon growth [103]. Importantly, NUMA1 was found to interact with ribosomal RNAs and to be involved in transcription of ribosomal DNA [101].

Finally, the *RBM22* gene was the only RBP we found upregulated by PCB 153. It is a splicing factor that participates during the activation and catalytic phases of the spliceosome cycle. Experimental depletion of RBM22 has been shown to impair mitotic and differentiation processes [104], and to trigger apoptosis as well [105]. However, some of these effects are independent of its splicing role. Interestingly, overexpression of RBM22 is correlated with diverse cancers, such as colon cancer [105] and glioblastoma [106].

Motif enrichment analysis identified CELF2, NUMA1, PRPF8, and RBM22 as candidate RBPs whose binding motifs are overrepresented in genomic regions associated with candidate DASEs. Given the known involvement of these proteins in RNA metabolism, spliceosome function, neuronal biology, or cellular stress responses, these findings provide a biologically plausible framework for future mechanistic investigation. However, motif enrichment alone cannot establish direct RBP occupancy or functional regulation. To minimize false-positive interpretation, RBP motifs were not considered as standalone evidence of RBP activity. Instead, they were interpreted only as supportive candidate-prioritization signals, particularly when occurring in positional proximity to DASE-associated regions and when consistent with the known RNA-processing or spliceosome-related functions of the corresponding proteins. In this context, CELF2, PRPF8, and RBM22 may represent more directly interpretable splicing-related candidates, whereas NUMA1 should be regarded as a less canonical and more indirect signal requiring additional validation. Future studies integrating CLIP/eCLIP datasets, RBP perturbation experiments, RNA immunoprecipitation, and targeted splicing assays will be required to determine whether these RBPs directly contribute to PCB 153-associated splicing changes.

Taken together, these data suggest that candidate splicing alterations may represent an additional regulatory layer associated with PCB 153 exposure. Given the highly specialized and tightly regulated splicing landscape of neuronal cells, even subtle perturbations could be biologically relevant; however, their functional consequences must be tested experimentally before definitive mechanistic conclusions can be drawn.

Another important consideration concerns the experimental model. RA-differentiated SH-SY5Y cells are widely used in neurotoxicity and neurobiology studies because they acquire neuron-like features and provide a reproducible human cellular system. Nevertheless, SH-SY5Y cells are neuroblastoma-derived and do not fully recapitulate the molecular identity, maturation state, cellular diversity, or tissue architecture of human neurons in vivo. Therefore, the candidate PCB 153-associated splicing alterations reported here should be validated in more physiologically relevant systems, including human iPSC-derived neurons, neural progenitor cells, mixed neuronal–glial cultures, or brain organoids.

### Study Strengths and Limitations

This study provides an exploratory computational analysis of previously generated RNA-seq data and identifies a prioritized set of candidate DASEs and candidate RBPs potentially associated with PCB 153 exposure in RA-differentiated SH-SY5Y cells. Moreover, RA-differentiated SH-SY5Y cells do not fully recapitulate normal neuronal biology, particularly in the context of neurodevelopmental processes. Although SH-SY5Y cells are widely used as a neuron-like model in neurobiology and neurodegenerative disease research [107,108], they derive from a human neuroblastoma and therefore retain tumor-related features, intrinsic heterogeneity, and differentiation-protocol-dependent phenotypes that may differ from those of mature primary neurons. These aspects limit their suitability for modeling complex neurodevelopmental events, including lineage specification, neuronal maturation, synaptogenesis, network formation, and interactions among multiple neural cell types [108]. In this context, the present findings should be interpreted as candidate molecular alterations identified in a simplified neuronal-like in vitro system, rather than as direct evidence of PCB 153 effects on human neurodevelopment. This limitation is particularly relevant for genes or pathways associated with neuronal differentiation, axonal guidance, synaptic organization, or developmental vulnerability. Future studies should therefore validate the prioritized DASEs, retained intron events, and RBP-related mechanisms in more physiologically relevant systems, including primary neuronal cultures, human iPSC-derived neurons, and, when feasible, 3D neural models or brain organoids [109,110]. These models may better capture aspects of human neuronal maturation, cellular heterogeneity, three-dimensional architecture, and developmental neurotoxicity-relevant phenotypes.

This study provides an exploratory computational re-analysis of previously generated RNA-seq data and identifies a prioritized set of candidate AS gene events and RBP-related regulatory hypotheses potentially associated with PCB 153 exposure in RA-differentiated SH-SY5Y cells. The strength of the study is the integration of differential expression analysis, r-MATS-based splicing detection, disease enrichment, RI/PTC prediction, and RBP motif enrichment within a single analytical framework. This approach enabled the prioritization of candidate genes and splicing events with potential relevance to neurodevelopmental disease annotations.

However, several limitations must be acknowledged. First, the analysis was based on two biological replicates per condition, which limits statistical power and reduces the generalizability of the findings. Although stringent filtering criteria were applied, including FDR correction, ΔPSI thresholds, and splice-junction coverage filtering, the identified DASEs should be considered candidate for events requiring validation in independent biological replicates. Moreover, multiple quality-control procedures were performed to assess data robustness. PCA, sample-to-sample distance analyses, dispersion diagnostics, MA plots, and Cook’s distance assessments consistently demonstrated high concordance between biological replicates and did not reveal any outlier samples. Therefore, our results, based on transcriptional patterns, were supported by the overall quality and consistency of the dataset. Second, no RT-PCR/RT-qPCR or protein-level validation was performed in the present study. Therefore, RI/PTC consequences, predicted NMD susceptibility, and potential effects on protein isoform production remain in computational predictions. Third, RBP motif enrichment does not demonstrate direct RBP binding or functional regulation, as motif accessibility depends on RNA structure, cellular context, RBP abundance, and competing RNA-protein interactions. Fourth, the use of TruSeq RNA Exome libraries may introduce capture-related coverage bias that can affect splice-junction quantification, although coverage-based filtering was applied to reduce low-confidence events. Fifth, RA-differentiated SH-SY5Y cells represent a useful neuron-like model for exploratory neurotoxicity studies but remain neuroblastoma-derived and cannot fully reproduce primary neurons, developing brain tissue, or multicellular neural systems.

Therefore, the present findings should not be interpreted as experimentally validated splicing alterations, but rather as computationally prioritized candidate events requiring independent molecular confirmation. Future studies should validate selected DASEs by RT-PCR/RT-qPCR in independent biological replicates, assess transcript and protein consequences of representative RI/PTC events, and investigate candidate RBPs using CLIP/eCLIP integration, RBP perturbation, or RNA immunoprecipitation approaches. Validation in iPSC-derived neurons, neural progenitor cells, mixed neuronal–glial cultures, or brain organoids would also help determine whether the candidate splicing alterations identified here are conserved in more physiologically relevant human neural models. Future studies incorporating a dose–response and time-course designs would further clarify whether PCB 153-associated candidate splicing alterations are concentration-dependent, temporally regulated, and reproducible across independent neuronal models.

## 5. Conclusions

In conclusion, this exploratory RNA-seq splicing analysis suggests that exposure of RA-differentiated SH-SY5Y cells to a sub-cytotoxic concentration (5 µM) of PCB 153 is associated with candidate alterations in AS and differential expression of RBP-encoding genes. The identified DASEs were enriched in genes annotated to ID and related neurodevelopmental phenotypes, while RI/PTC prediction and RBP motif enrichment analyses nominated selected transcripts and RBPs for future validation. Importantly, these findings remain computational and hypothesis-generating. Overall, the study provides a prioritized framework for investigating post-transcriptional mechanisms potentially involved in PCB 153 neurotoxicity and highlights the need for independent experimental validation in more physiologically relevant neuronal models.

These findings support the inclusion of post-transcriptional regulation among the molecular processes to be investigated in future environmental neurotoxicology studies.

## Figures and Tables

**Table 1:** Enrichment analysis of genes with candidate PCB 153-associated differential alternative splicing (DASE).

Enrichment Analysis	N. Enriched Entries	Top Enriched Term	Top Enriched Term’s Genes
Molecular Function	5	Histone H4K20 Methyltransferase Activity (GO:0042799)	*NSD1*, *KMT5B*
DisGeNET Diseases	68	Intellectual Disability	*ABCD4*, *KMT2A*, *DCTN1*, *HTRA2*, *VLDLR*, *AFF4*, *OPA1*, *FLAD1*, *NSD1*, *KMT5B*, *ACADM*, *TXNL4A*, *CTSA*, *RUBCN*, *ADSL*, *STRADA*, *UROS*, *TINF2*, *LAMB1*, *GDAP1*, *POMGNT1*, *CLK2*, *DCX*, *RAB3GAP2*, *TAF1*, *FGFR1*

GO, KEGG, and DisGeNET analyses revealed no significant enrichment for biological processes, cellular components, or KEGG pathways. Significant enrichment was limited to GO molecular function, with histone H4K20 methyltransferase activity (GO:0042799) (NSD1, KMT5B) as the top term. Disease association analysis showed a strong over-representation of intellectual disability, supported by multiple neurodevelopment-related genes.

**Table 2:** PTC analysis of ID-associated RI events in PCB 153 vs. CTRL.

Gene	Genomic Coordinates (hg38)	ΔPSI	Reading Frame	Position of First PTC; Distance Relative to the Last Exon–Exon Junction	No. PTCs
*CTSA*	chr20:45892323-45892397(+)	−0.357	1	4; 70	1
*TINF2*	chr14:24240330-24240418(−)	−0.208	3	2; NA	1
*POMGNT1*	chr1:46194651-46194843(−)	−0.190	2	60; 131	1
	chr1:46194961-46195810(−)	−0.241	1	37; 812	17
*ADSL*	chr22:40361635-40362980(+)	−0.121	3	59; 1286	36
*FGFR1*	chr8:38428093-38428351(−)	−0.175	2	57; 201	1
*HTRA2*	chr2: 74531702-74531855(+)	−0.138	2	33; 120	1
*DCTN1*	chr2:74368131-74368727(−)	−0.185	1	49; 547	10
*RAB3GAP2*	chr1:220167399-220167501(−)	0.197	2	93; 9	1

RI events were filtered for genes associated with ID. For each RI event, the correct reading frame was manually identified by inspecting the corresponding genomic region in IGV. The sequence of the RI introduced by the ASE was translated, and any in-frame PTCs (TAA, TAG, TGA) resulting from the intron retention were reported, along with the ΔPSI value of the corresponding RI event. In addition, the number of PTCs predicted, the position of the first PTC within the RI sequence (counting from the upstream exon–intron pre-mRNA junction), and the distance relative to the last exon–exon junction, were annotated. NA stands for not applicable.

## Data Availability

All data generated and analyzed in this study are available in the NCBI Sequence Read Archive (SRA) under the BioProject accession number PRJNA1378135. All custom scripts used for data processing and figure generation are publicly available at: https://github.com/marialui/PCB153_VS_CTRL_splicing_analysis, accessed on 26 March 2026.

## Supplementary Materials

The following supporting information can be downloaded at: https://www.mdpi.com/article/10.3390/genes17060692/s1, Figure S1: Principal component analysis of variance-stabilized expression data; Figure S2: DESeq2 dispersion plot; Figure S3: MA plot of differential expression; Figure S4: Cook’s distance plot; Figure S5: Heatmap of gene–disease associations; Table S1: RBP DEGs; Table S2: ID-associated DASEs; Table S3: Enrichment analysis; Table S4: PTC analysis; Table S5: RBP motif enrichment analysis.

## Abbreviations

The following abbreviations are used in this manuscript:

AD	Alzheimer’s disease
ALS	Amyotrophic lateral sclerosis
AS	Alternative mRNA splicing
ASE	Alternative splicing event
A3SS	Alternative 3′ splice site
A5SS	Alternative 5′ splice site
CTRL	Control sample
DASE	Differentially alternative splicing event
DEG	Differentially expressed gene
EJC	Exon junction complex
FDR	False discovery rate
FGF	Fibroblast growth factor
GO	Gene ontology
ID	Intellectual disability
MXE	Mutually exclusive exons
NMD	Nonsense-mediated decay
PCB	Polychlorinated biphenyl
PD	Parkinson’s disease
PSI	Percent spliced in
PTC	Premature termination codon
RA	Retinoic acid
RI	Retained intron
RBP	RNA-binding proteins
RMATS	Replicate Multivariate Analysis of Transcript Splicing
SE	Skipped exon
snRPs	Small nuclear ribonucleoproteins
UTR	Untranslated region
