# Supplementary figures and images for: Environmental Pollutant PCB 153 Is Associated with Candidate Alternative Splicing Alterations in Intellectual Disability-Associated Genes: An Exploratory RNA-Seq Splicing Analysis in a Neuronal Model
Source: Genes (Basel). 2026 Jun 13;17(6):692. doi: 10.3390/genes17060692 (PMC13300575; doi:10.3390/genes17060692)

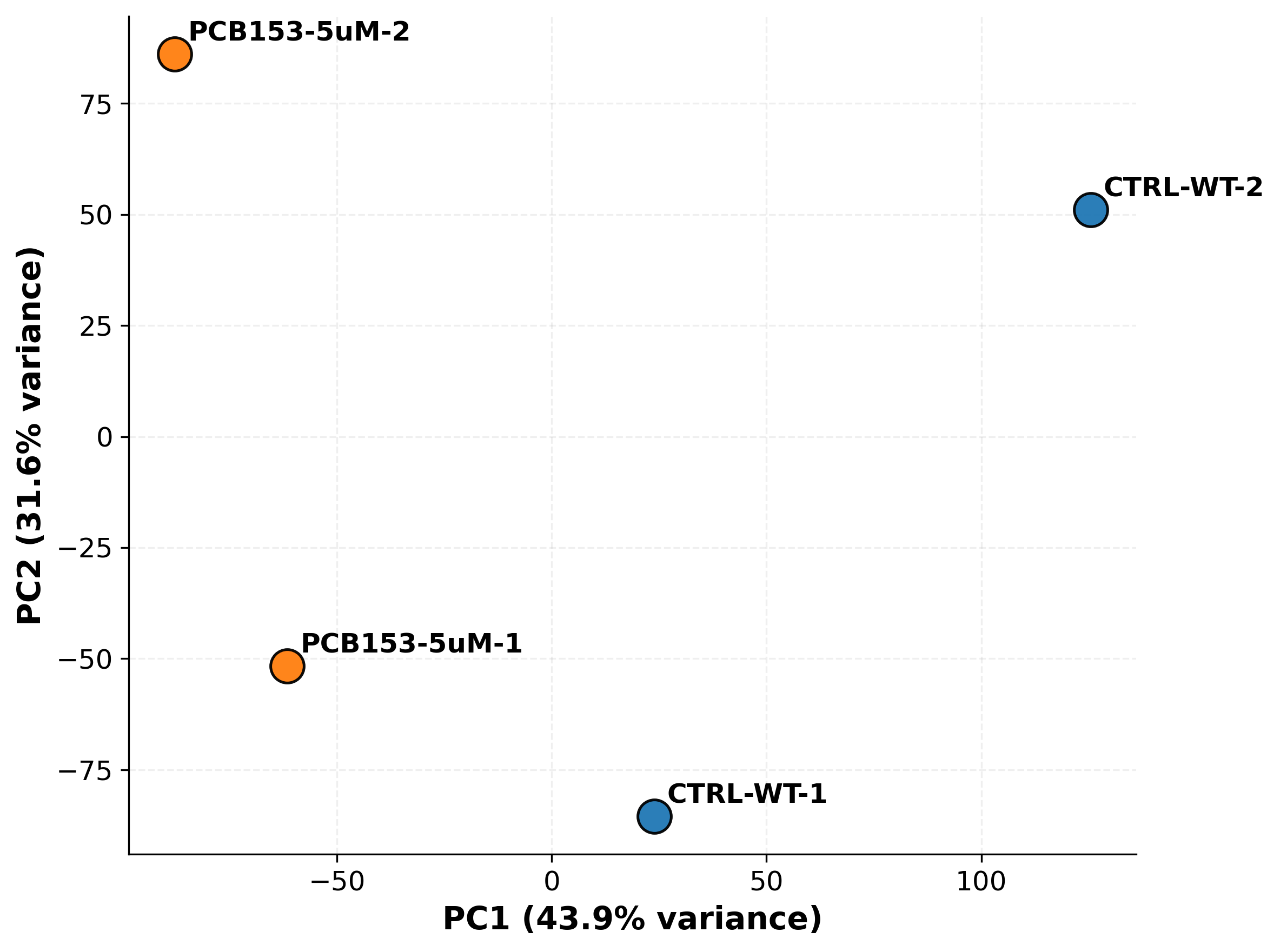

Supplement: Supplementary file 1 [file genes-17-00692-s001.zip › Figure S1.png]

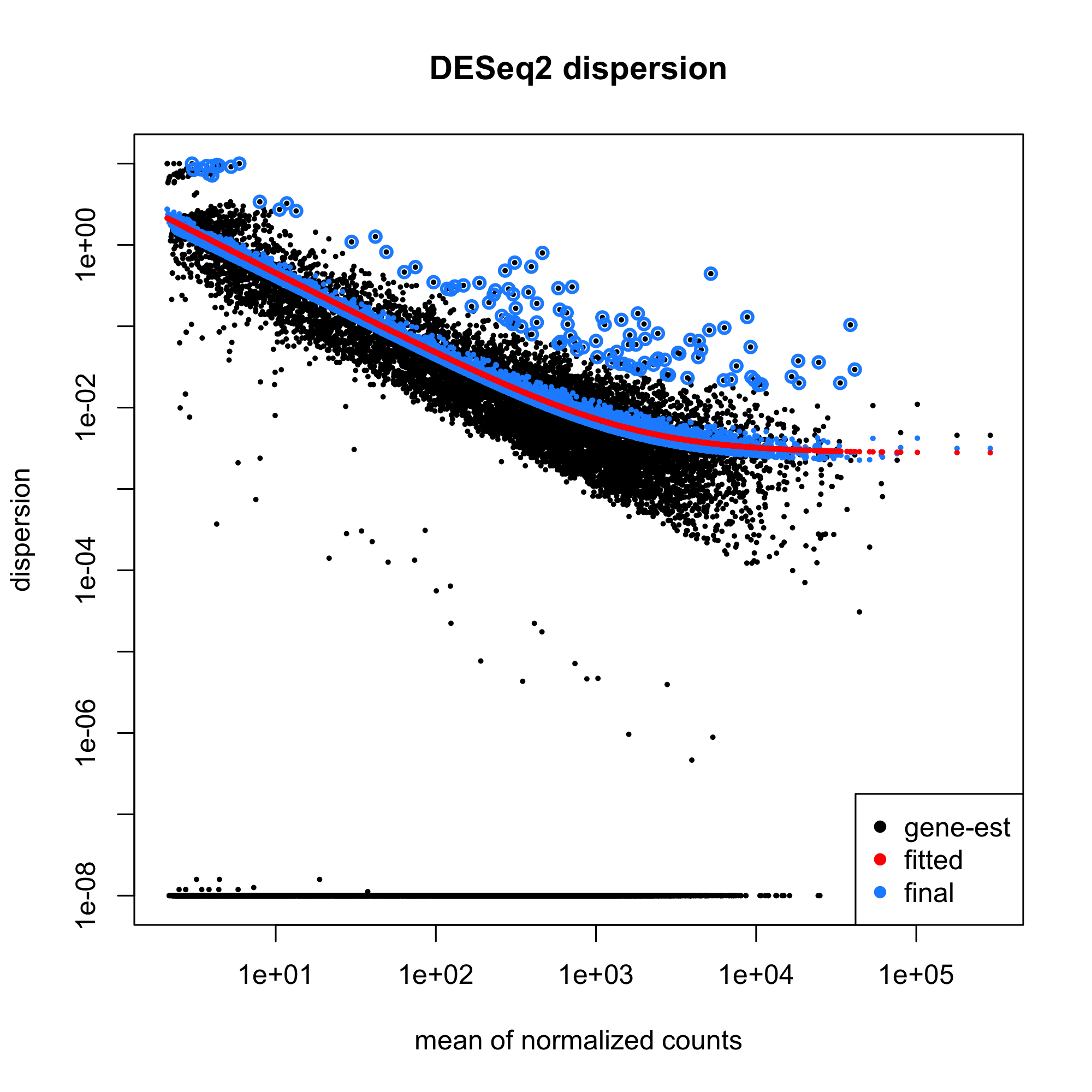

Supplement: Supplementary file 1 [file genes-17-00692-s001.zip › Figure S2.png]

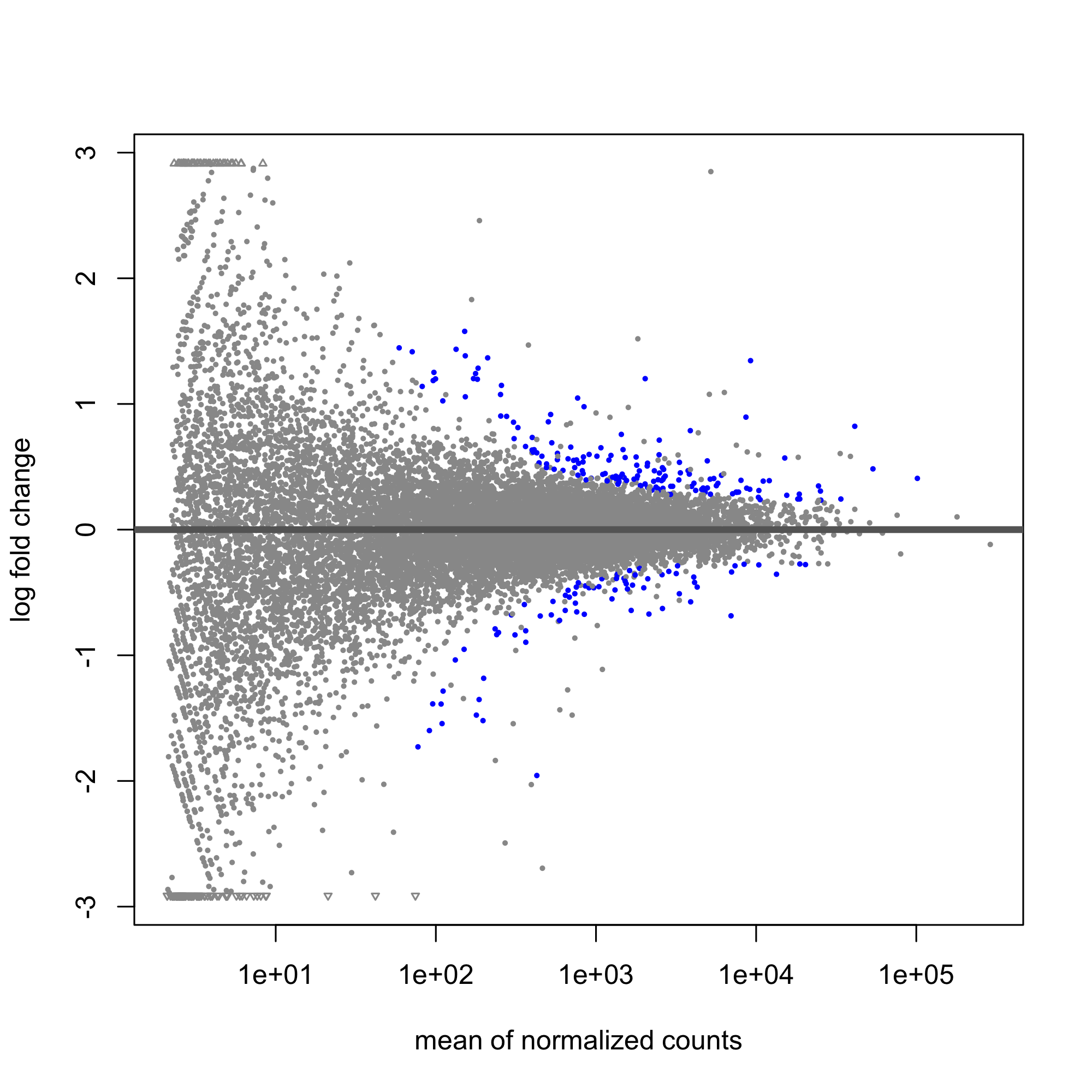

Supplement: Supplementary file 1 [file genes-17-00692-s001.zip › Figure S3.png]

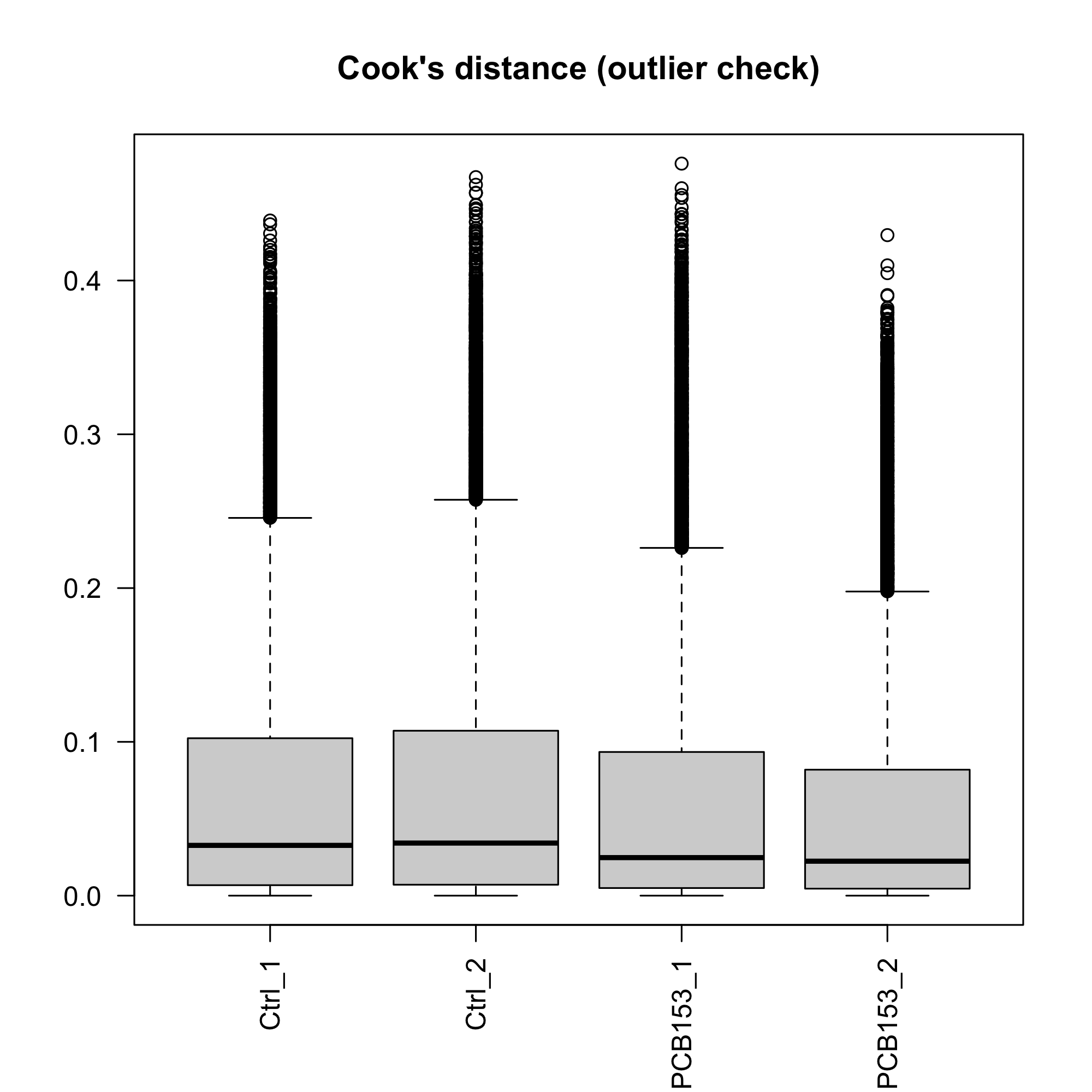

Supplement: Supplementary file 1 [file genes-17-00692-s001.zip › Figure S4.png]

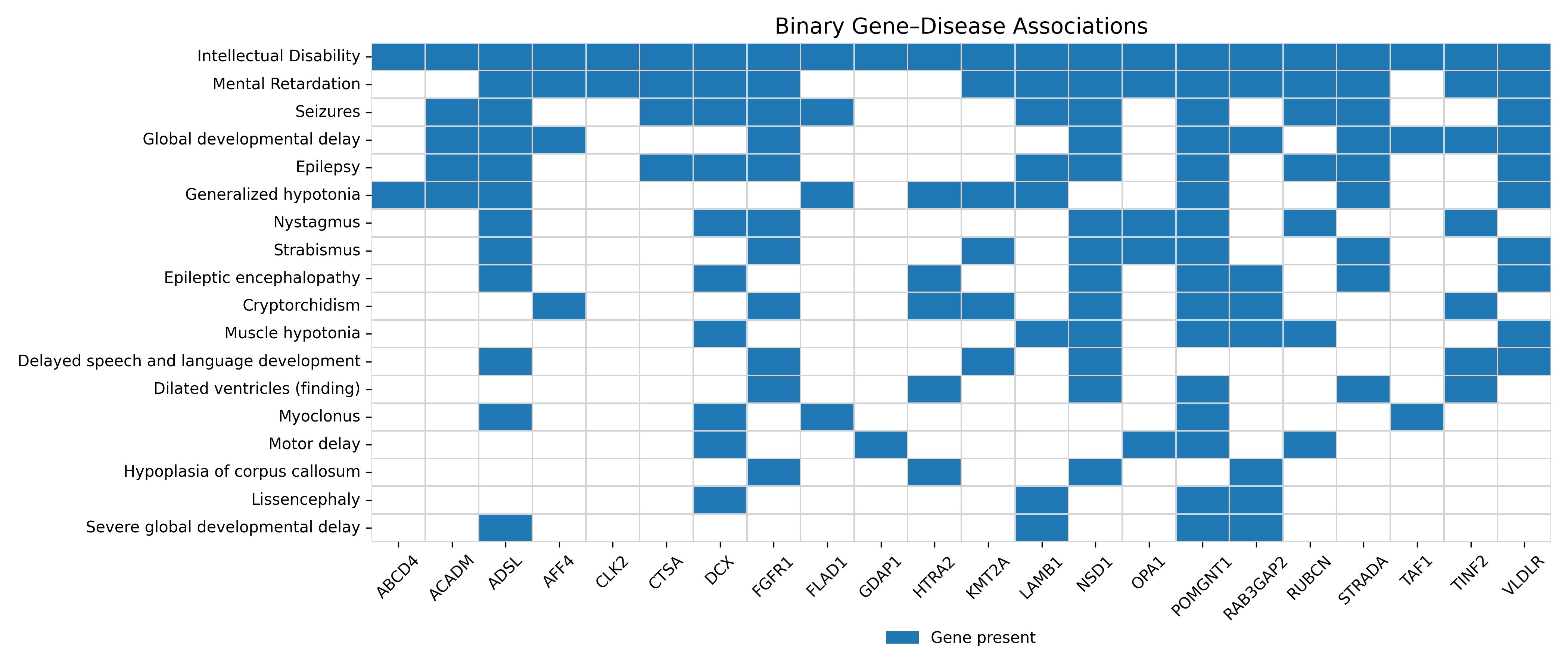

Supplement: Supplementary file 1 [file genes-17-00692-s001.zip › Figure S5.png]
